# [Retracted] An altered expression of genes involved in the regulation of ion channels in atrial myocytes is correlated with the risk of atrial fibrillation in patients with heart failure

**DOI:** 10.3892/etm.2026.13241

**Published:** 2026-07-13

**Authors:** Mei Gao, Jiangrong Wang, Zhongsu Wang, Yong Zhang, Hui Sun, Xinxing Xie, Yinglong Hou

Exp Ther Med 5:1239–1343, 2013; DOI: 10.3892/etm.2013.949

Following the publication of this paper, it was drawn to the Editor’s attention by a concerned reader that western blot data featured in Fig. 2A on p. 1241 were strikingly similar to data that had already appeared in different form in Fig. 3C of another article written by different authors at different research institutes in the journal *Proceedings of the National Academy of Sciences.*

In view of the fact that the abovementioned data had already apparently been published before this article was submitted to *Experimental and Therapeutic Medicine*, the Editor has decided that this paper should be retracted from the Journal. The authors were asked for an explanation to account for these concerns, but the Editorial Office did not receive a reply. The Editor apologizes to the readership for any inconvenience caused.

